# Ameliorative action of eugenol on nitrate induced reproductive toxicity in male rats

**DOI:** 10.1016/j.toxrep.2024.101702

**Published:** 2024-07-30

**Authors:** Sulanaikanahalli Vadyappa Rajini, Halugudde Nagaraja Sarjan, Shivabasavaiah

**Affiliations:** a Department of Zoology, ST Joseph’s University, Bangalore, Karnataka, India; b Department of Studies in Zoology, Manasagangotri, University of Mysore, Mysore, Karnataka, India

**Keywords:** Abnormality, Eugenol, Motility, Nitrate, Nitrite, Testosterone

## Abstract

There is a great concern for studies to prevent nitrate (NO_3_) induced male reproductive toxicity as it might lead to infertility. Therefore, the study was aimed to investigate the ameliorative effects of eugenol on NO_3_ induced male reproductive toxicity in wistar rats. Adult male rats were randomly divided into five groups (n=5). The first group was served as control, the second and third group of rats were treated with 100 mg/kg bw of sodium nitrate (NaNO_3_) and NO_3_ contaminated ground water respectively. The fourth and fifth group of rats were orally intubated with eugenol (100 mg/kg bw) and then exposed to NaNO_3_ and NO_3_ contaminated ground water respectively. The treatment was continued for 52 days. Nitrate exposure significantly decreased the sperm motility, testicular 3-beta-hydroxysteroid dehydrogenase activity, serum concentration of testosterone, activities of superoxide dismutase and catalase in testis and spermatozoa and different categories of germ cells in stage VII of spermatogenesis. Further, there was significant increase in sperm abnormality and levels of nitrite (NO_2_) and malondialdehyde in testis and spermatozoa of NO_3_ treated rats. In addition, NO_3_ exposure distorted the histological architecture of seminiferous tubules of testis. It was established that NO_3_ induced high production of NO_2_ affected spermatogenesis, steroidogenesis and sperm motility. However, in the present study, pretreatment of eugenol prevented NO_3_ induced reproductive alterations by decreasing the level of NO_2_. These findings clearly showed the protective action of eugenol against NO_3_ induced oxidative stress in male reproductive system.

## Highlights

• Nitrate induced high production of nitrite and oxidative stress affected spermatogenesis, steroidogenesis and sperm motility.
• Eugenol from *Syzygium aromaticum* prevented NO_3_ induced reproductive toxicity in male wistar rats.
• Eugenol pretreatment prevented excess production of nitrite and thereby protected the male reproductive system.

## Graphical Abstract

**Figure:**
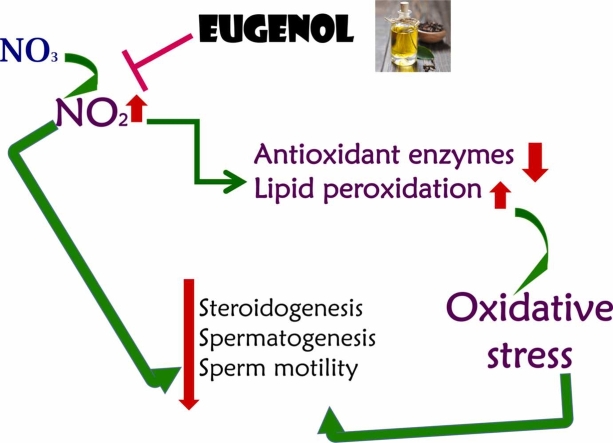

## 1 Introduction

Globally, the nitrate (NO_3_) content in ground water is increasing due to anthropogenic activities, as well as nitrogenous fertilizers [4]. Studies have been reported that high level of NO_3_ adversely affect reproductive system of rats, rabbits and mice [8], [11], [10], [1]. Decrease in sperm motility and sperm count and increase in sperm abnormality, disturbed spermatogenesis, degeneration of Leydig cells, decrease in the population of spermatogonial cells and loss of germ cells are the alterations observed in animals under NO_3_ treatment [1], [10], [11], [41], [7], [8]. Further, NO_3_ exposure reduces the concentration of serum testosterone and the activity of 3-beta-hydroxysteroid dehydrogenase (3β-HSD) in rats and mice [41], [8]. In addition, it has been reported that NO_3_ induced increased production of nitrite (NO_2_) and further nitric oxide (NO) induces oxidative stress and thereby affect male reproduction and reproductive performances [27]. In this regard, attempts have been undertaken to prevent NO_3_ induced reproductive complications in different animal models [1], [10], [11], [20]. In recent years, more attention has been focused on the use of herbal medicines and their derivatives in treatment of conditions related to oxidative stress [29] as herbal media has least or no side effects.

A study reported that supplementation of vitamin C, vitamin E with selenium and the probiotic prevented sodium nitrate (NaNO_3_) induced decrease in plasma testosterone concentration, alterations in testis histology, decrease in fertility and reduced number and weight of offspring produced in rabbits [10]. Further, extract of *Chlorella vulgaris* prevented sodium nitrite (NaNO_2_) induced decreased level of follicle-stimulating hormone (FSH), testosterone, sperm count, sperm motility and sperm viability, altered activities of testicular antioxidant enzymes and histological architecture in male albino rats [20]. Similarly, oral administration of walnut oil has reduced the oxidative stress and improved the spermatogenesis in NaNO_2_ exposed rats [1]. In addition, oral administration of curcumin and turmeric prevented NaNO_3_ induced reduction in epididymal sperm number, weights of testis and epididymis, concentration of testosterone and dehydroepiandrosterone and testicular 3β-HSD activity in rats [11].

Eugenol is one such natural phenolic and aromatic compound from clove oil of *Syzygium aromaticum* [53] and essential oils of plants from the families *viz.,* Lamiaceae, Lauraceae, Myrtaceae and Myristicacea. Several studies have proved the various antioxidant, anti-inflammatory, analgesic, anti-allergic, anti-mutagenic and anti bacterial properties of eugenol [13], [17], [18], [30]. In addition, it has been reported that eugenol inhibits reactive oxygen species (ROS) and NO production in human neutrophils which were stimulated by phorbol 12- myristate 13 acetate or hydrogen peroxide (H_2_O_2_) [53]. Further, studies have shown ameliorative action of eugenol (100 mg/kg bw) in preventing the nephrotoxicity which was caused by gentamicin [47], chromium [12] and streptozotocin [22] in rats. The exposure to potassium dichromate (K_2_Cr_2_O_7_) caused significant decrease in the activities of renal reduced glutathione (GSH) and superoxide dismutase (SOD) and increase in renal malondialdehyde (MDA) levels. However, pretreatment of eugenol prevented K_2_Cr_2_O_7_ induced alterations in renal antioxidant system of rats and suggested its antioxidant property [12]. It has been proved that oral administration of eugenol for 10 days reduced the hepatotoxicity caused by iron [44] and carbon tetrachloride [35] in rats. Pretreatment of eugenol for 15 days prevented the liver injury induced by thioacetamide in adult male rats [57]. In addition, eugenol administration to diabetic rats improved body weight and hepatic glycogen content and also improved the key enzymes of glucose metabolism [49]. Few studies are focused on ameliorative action of eugenol on reproductive toxicity. Administration of eugenol improved the erectile dysfunction in diabetic rats [56]. Further, intra-peritoneal administration of 100 mg/kg bw of eugenol prevented cisplatin induced oxidative stress and apoptosis in rat testis. Exposure to cisplatin caused increase in the testicular lipid peroxidation and caspase-3 levels and decrease in the activities of testicular antioxidant enzymes. However, lipid peroxidation decreased and antioxidant enzyme activities were increased in the eugenol treated group [6]. These results further confirmed the antioxidant property of eugenol. It has been reported that, eugenol improves the level of testosterone, luteinizing hormone, FSH and reduces oxidative stress in morphine withdrawn rats thereby increases reproductive function [32], [33].

There are few studies on protective action of eugenol on various systems of the body under the exposure of different chemicals. However, the studies related to the efficacy of eugenol in preventing NO_3_ induced male reproductive toxicity are lacking. Few studies clearly showed the fact that the ameliorative action of eugenol is due to its antioxidant property. Hence, the study aims to investigate the antioxidant action of eugenol in preventing NaNO_3_ and NO_3_ contaminated ground water induced reproductive toxicity in male rats.

## 2 Materials and methods

### 2.1 Animals

Adult male wistar rats weighing about 160–200 g were procured from Central Animal Facility, University of Mysore, Mysore, India. The Animals were maintained under standard laboratory conditions of relative humidity (37±10 %), temperature (25±2^0^ C) and 12 h light/12 h dark cycles. The animals were fed with normal laboratory diet and clean drinking water. Experimental procedures were followed after the approval by the Institutional Animal Ethics Committee of University of Mysore, India (Reference number: UOM/IAEC/04/2018).

### 2.2 Experimental design

Adult male rats were randomly divided into five groups of five animals each (n=5). The first group of rats were served as control and were maintained without any disturbances. The second group of rats was orally intubated with 100 mg/kg bw/1 mL/rat of NaNO_3_. The third group of rats was allowed to drink NO_3_ contaminated ground water. The fourth group of rats was pretreated with 100 mg/kg bw of eugenol and then exposed to NaNO_3_. However, the fifth group of rats was pretreated with 100 mg/kg bw of eugenol and then allowed to drink NO_3_ contaminated ground water. A preliminary experiment was conducted by using different concentration of eugenol *viz.,* 1 mg/kg bw, 10 mg/kg bw and 100 mg/kg bw, wherein 100 mg/kg bw of eugenol was found to be effective, hence the same concentration was used for the present study. The treatment period of the current experiment was based on the duration of spermatogenic cycle of rats, a period of 52 days [21]. Hence, the effect of NO_3_ on spermatogenesis, steroidogenesis and sperm motility was assessed by considering one spermatogenic wave of rats. In addition, before conducting the current experiment, a pilot study was performed to demonstrate the effects of different doses of NaNO_3_ (10, 100, 500 and 1000 mg/kg bw) on male reproductive system, pancreas, kidney and liver of rats for a duration of 52 days. The study clearly demonstrated the toxic effects of NO_3_ on both reproductive system and other organs of the body [42]. Therefore, 52 days of treatment period was considered for the present experiment. After the treatment period, the rats were euthanized and the percentage change in body weight and relative organ weight were noted. The epididymal sperm suspension was used for the analysis of sperm motility and abnormality. One of the testis was used for histological studies and the remaining one was used for biochemical estimations. Serum was collected from the blood and was used for the estimation of testosterone concentration.

### 2.3 Collection of ground water

The NO_3_ contaminated ground water was collected from Kadakola town of Mysore district of Karnataka, India. Water sample was collected from hand pumps and stored in polypropylene bottles. The concentration of NO_3_ in the water sample was estimated by copper cadmium reduction method and it was found to be 141.47 mg/L [50]. The concentration of trace elements in NO_3_ contaminated ground water was estimated and the values were in permissible range (Table 1).

**Table 1:** Concentration of different trace elements in NO_3_ contaminated ground water.

SL. No	Trace elements	Concentration (mg/L)
1	Calcium	65.6
2	Chloride	218.76
3	Magnesium	38.09
4	Boron	BDL (<0.1)
5	Sulphate	51.97
6	Fluoride	0.20
7	Iron	BDL(<0.02)

Note: BDL- Below Detection Level.

### 2.4 Parameters studied

#### 2.4.1 Body and relative organ weight

The initial body weight of individual rat was noted before the commencement of experiment. The final body weight and the weight of testis, epididymis, vas deferens and seminal vesicle were noted during the autopsy. The relative weight of an organ was calculated.

### 2.5 Isolation of spermatozoa

The spermatozoa were isolated from cauda epididymis of rats soon after the autopsy. The epididymis was minced in 1 mL of phosphate buffered saline (pH-7.2) and was filtered through muslin cloth. The obtained sperm suspension was used for the analysis of sperm motility and abnormality.

#### 2.5.1 Sperm motility

Immediately after obtaining the sperm suspension, few drops of the suspension was placed on a clean glass slide and observed under the bright field microscope. A total of hundred spermatozoa was considered in a microscopic field for the counting. The number of spermatozoa showing progressive motility out of hundred spermatozoa was noted down. The spermatozoa exhibiting non-progressive movement and immotile spermatozoa were not considered for the motility count. The procedure was repeated for other two different microscopic fields. The mean count of three microscopic fields was calculated and expressed in percentage of motile spermatozoa [46].

#### 2.5.2 Sperm abnormality

The sperm suspension was mixed with a drop of 1 % aqueous eosin stain and was placed on a glass slide. Gently, an uniform smear was made on the glass slide and it was air dried. A total of one thousand spermatozoa were observed under 40X magnification of bright field microscope. The number of spermatozoa showing different types of sperm abnormality *viz.,* coiled tail, bent tail, hairpin tail, amorphous head, pyriform head and banana head were counted. The percentage of abnormal spermatozoa was calculated for the total of one thousand spermatozoa [37], [54].

### 2.6 Steroidogenic activity

The activity of the testicular 3β-HSD was measured as per the procedure of Shivanandappa and Venkatesh [48].

### 2.7 Serum concentration of testosterone

The serum concentration of testosterone was estimated by following the procedure of testosterone diagnostic ELISA kit manufactured by CALBIOTEC Esdoomiaan 13,395 IDB Maam, Netherland, Germany. The concentration of serum testosterone was expressed as ng/mL.

### 2.8 Biochemical estimations

The activity of SOD in testis and spermatozoa was assayed by following the method of Marklund and Marklund [31] (sensitivity 16 unit/mg protein).

The activity of catalase (CAT) in testis and spermatozoa was assayed according to the method of Aebi [3] (sensitivity 0.25–4nmol/min/mL).

The concentration of MDA in testis and spermatozoa was analyzed as per the method of Ohkawa et al. [38] (sensitivity 4 nmol/mg protein).

The concentration of NO_2_ in testis and spermatozoa was estimated by classic colorimetrical Griess reaction as per the procedure of Avdagic et al. [2].

### 2.9 Histology and study of spermatogenesis

Testes were placed in Bouin’s fixative. The fixed tissues were sequentially subjected to dehydration, clearing and embedded in paraffin wax. About 5µ thick tissue sections were taken and stained with haematoxylin and eosin. The histological alterations were observed under light microscope and were photographed. The number of each category of germ cells in stage VII of seminiferous epithelium cycle, i.e. type A spermatogonia, preleptotene spermatocytes, midpachytene spermatocytes, round and elongated spermatids were counted from ten round tubular cross sections of testis of each rat [14], [16]. All counts of the germ cells were converted to true counts by the formula,

$$\mathrm{True}\;\mathrm{counts}=\frac{\left(\mathrm{crude}\;\mathrm{count}\times \mathrm{section}\;\mathrm{thickness}\right)}{\left(\mathrm{section}\;\mathrm{thickness}+\mathrm{nuclear}\;\mathrm{diameter}\;\mathrm{of}\;\mathrm{germ}\;\mathrm{cells}\right)}$$

### 2.10 Statistical analysis

The mean ± standard error of each parameter was computed by considering the data of 5 rats per group. The mean values of each parameter of different groups were compared using one way analysis of variance followed by Duncan’s multiple range test and judged significant if p < 0.05.

## 3 Results

### 3.1 The percentage change in body weight and relative weights of testis, epididymis, vas deferens and seminal vesicles

There was significant increase in percentage gain in body weight and decrease in relative weights of testis, epididymis, seminal vesicle and vas deferens in NaNO_3_ and NO_3_ contaminated ground water treated rats compared to that of controls. However, percentage gain in body weight and relative weights of testis, epididymis, seminal vesicle and vas deferens of eugenol pretreated NO_3_ exposed rats were similar to that of NO_3_ treated rats (Table 2).

**Table 2:** Body weight and relative weight of testis, epididymis, vas deferens and seminal vesicles of male rats.

Groups	Mean % gain in body weight (g) ±SE	Mean relative weight (mg/100 g body weight) ± SE			
		Testis	Epididymis	Vas deferens	Seminal vesicle
**Control**	25.41±5.15^a^	1.37±0.05^a^	0.57±0.10^a^	0.13±0.01^a^	0.53±0.04^a^
**NaNO**_**3**_**(100 mg/kg bw)**	61.59±8.38^b^	1.26±0.01^ab^	0.32±0.02^b^	0.07±0.00^b^	0.38±0.02^b^
**NO**_**3**_**contaminated ground water**	69.15±12.28^b^	1.05±0.02^c^	0.33±0.00^b^	0.07±0.00^b^	0.39±0.02^b^
**NaNO**_**3**_**+ Eugenol (100 mg/kg bw)**	49.10±10.94^ab^	1.16±0.06^bc^	0.34±0.01^b^	0.07±0.00^b^	0.31±0.00^b^
**GW+ Eugenol (100 mg/kg bw)**	52.04±6.94^ab^	1.25±0.03^ab^	0.39±0.00^b^	0.09±0.01^b^	0.34±0.02^b^
**ANOVA F value** **df = (5, 24)**	3.308 P<0.03	7.75 P<0.001	4.52 P<0.009	9.23 P<0.0001	9.36 P<0.0001

**Note**: Mean values with same superscript letters in the given column are not significantly different, whereas those with different superscript letters are significantly (P < 0.05) different as judged by Duncan’s multiple test df: degree of freedom, NaNO_3_- sodium nitrate, GW – NO_3_ contaminated ground water.

### 3.2 Sperm motility and sperm abnormality

There was significant reduction in sperm motility and increase in sperm abnormality in NaNO_3_ and NO_3_ contaminated ground water treated rats compared to that of control rats. However, the sperm motility of eugenol pretreated NO_3_ exposed rats did not differ from control group. The number of abnormal spermatozoa in eugenol pretreated NO_3_ exposed rats was significantly lower than NO_3_ treated rats and higher than control rats (Table 3).

**Table 3:** Sperm motility and sperm abnormality of male rats.

Groups	Mean % of motile spermatozoa ± SE	Mean number of abnormal spermatozoa/1000 spermatozoa ± SE
**Control**	99.00±1.00^a^	9.20±1.56^a^
**NaNO**_**3**_**(100 mg/kg bw)**	47.20±8.99^b^	32.40±2.61^d^
**NO**_**3**_**contaminated ground water**	45.60±2.11^b^	24.80±1.93^c^
**NaNO**_**3**_**+ Eugenol (100 mg/kg bw)**	77.20±6.49^a^	16.00±1.51^b^
**GW+ Eugenol (100 mg/kg bw)**	85.60±11.49^a^	18.60±0.97^b^
**ANOVA F value** **df = (5, 24)**	10.77 P<0.0001	23.86 P<0.0001

**Note**: Mean values with same superscript letters in the given column are not significantly different, whereas those with different superscript letters are significantly (P < 0.05) different as judged by Duncan’s multiple test df: degree of freedom, NaNO_3_- sodium nitrate, GW – NO_3_ contaminated ground water.

### 3.3 Activities of antioxidant enzymes and concentration of MDA

The activities of testicular and spermatozoa SOD and CAT were significantly reduced in NaNO_3_ and NO_3_ contaminated ground water treated rats compared to that of control rats. However, the activities of testicular and spermatozoa SOD and CAT in eugenol pretreated NO_3_ exposed rats were similar to that of control rats (Table 4).

**Table 4:** Activities of CAT and SOD of male rats.

Groups	Testis		Spermatozoa	
	Mean activity of CAT (nmol/mg/min) ± SE	Mean activity of SOD (U/mg protein) ± SE	Mean activity of CAT (nmol/mg/min) ± SE	Mean activity of SOD (U/mg protein) ± SE
**Control**	0.02±0.002^a^	0.56±0.02^a^	0.02±0.005^a^	0.42±0.01^a^
**NaNO**_**3**_**(100 mg/kg bw)**	0.007±0.002^b^	0.42±0.01^b^	0.004±0.002^b^	0.21±0.01^b^
**NO**_**3**_**contaminated ground water**	0.006±0.002^b^	0.42±0.03^b^	0.004±0.003^b^	0.24±0.02^b^
**NaNO**_**3**_**+ Eugenol (100 mg/kg bw)**	0.02±0.003^a^	0.53±0.01^a^	0.02±0.005^a^	0.39±0.005^a^
**GW+ Eugenol (100 mg/kg bw)**	0.02±0.003^a^	0.55±0.05^a^	0.02±0.005^a^	0.40±0.006^a^
**ANOVA F value** **df = (5, 24)**	10.37 P<0.0001	4.63 P<0.009	4.50 P<0.01	29.57 P<0.0001

**Note**: Mean values with same superscript letters in the given column are not significantly different, whereas those with different superscript letters are significantly (P < 0.05) different as judged by Duncan’s multiple test df: degree of freedom, NaNO_3_- sodium nitrate, GW – NO_3_ contaminated ground water.

There was significant increase in the concentration of MDA in testis and spermatozoa of NaNO_3_ and NO_3_ contaminated ground water treated rats compared to that of control rats. However, the concentration of MDA of testis and spermatozoa in eugenol pretreated NO_3_ exposed rats was similar to that of control rats (Table 5).

**Table 5:** Concentration of MDA of testis and spermatozoa in male rats.

Groups	Mean testicular MDA (nmol/mg protein) ± SE	Mean MDA of spermatozoa (nmol/mg protein) ± SE
**Control**	67.21±9.96^a^	76.35±6.37^a^
**NaNO**_**3**_**(100 mg/kg bw)**	153.18±10.44^b^	269.37±20.31^b^
**NO**_**3**_**contaminated ground water**	161.38±15.69^b^	366.15±44.45^c^
**NaNO**_**3**_**+ Eugenol (100 mg/kg bw)**	92.92±6.58^a^	97.57±9.41^a^
**GW+ Eugenol (100 mg/kg bw)**	86.64±7.73^a^	118.80±14.96^a^
**ANOVA F value** **df = (5, 24)**	16.02 P<0.0001	29.06 P<0.0001

**Note**: Mean values with same superscript letters in the given column are not significantly different, whereas those with different superscript letters are significantly (P < 0.05) different as judged by Duncan’s multiple test df: degree of freedom, NaNO_3_- sodium nitrate, GW – NO_3_ contaminated ground water.

### 3.4 Activity of testicular 3β-HSD and serum concentration of testosterone

There was significant decrease in the activity of testicular 3β-HSD and serum concentration of testosterone in NaNO_3_ and NO_3_ contaminated ground water treated rats compared to that of control rats. However, the activity of testicular 3β-HSD and serum concentration of testosterone were not significantly differed between eugenol pretreated NO_3_ exposed and control group of rats (Table 6).

**Table 6:** Activity of testicular 3β-HSD and serum concentration of testosterone in male rats.

Groups	Mean activity of testicular 3β-HSD (nmol/mg/min) ± SE	Mean serum concentration of testosterone (ng/mL) ± SE
**Control**	0.11±0.01^a^	4.12±1.01^a^
**NaNO**_**3**_**(100 mg/kg bw)**	0.05±0.00^b^	1.08±0.48^b^
**NO**_**3**_**contaminated ground water**	0.05±0.01^b^	1.09±0.10^b^
**NaNO**_**3**_**+ Eugenol (100 mg/kg bw)**	0.09±0.00^a^	3.16±0.41^a^
**GW+ Eugenol (100 mg/kg bw)**	0.09±0.01^a^	4.08±0.25^a^
**ANOVA F value** **df = (5, 24)**	5.85 P<0.003	7.75 P<0.001

**Note**: Mean values with same superscript letters in the given column are not significantly different, whereas those with different superscript letters are significantly (P < 0.05) different as judged by Duncan’s multiple test df: degree of freedom, NaNO_3_- sodium nitrate, GW – NO_3_ contaminated ground water.

### 3.5 Concentration of NO_2_ in testis and spermatozoa

The concentration of testicular and spermatozoa NO_2_ was significantly increased in NaNO_3_ and NO_3_ contaminated ground water treated rats compared to that of control rats. However, the concentration of NO_2_ in testis and spermatozoa of eugenol pretreated NO_3_ exposed rats was similar to that of control rats (Table 7).

**Table 7:** Concentration of NO_2_ in testis and spermatozoa of male rats.

Groups	Mean testicular NO_2_ (umol/g) ± SE	Mean NO_2_ of spermatozoa (umol/g) ±SE
**Control**	115.42±9.05^a^	77.60±3.02^a^
**NaNO**_**3**_**(100 mg/kg bw)**	193.46±5.33^b^	138.63±12.34^b^
**NO**_**3**_**contaminated ground water**	172.06±10.50^b^	127.00±12.19^b^
**NaNO**_**3**_**+ Eugenol (100 mg/kg bw)**	111.12±6.11^a^	93.74±8.54^a^
**GW+ Eugenol (100 mg/kg bw)**	135.35±5.03^a^	96.00±3.05^a^
**ANOVA F value** **df = (5, 24)**	20.71 P<0.0001	7.32 P<0.001

**Note**: Mean values with same superscript letters in the given column are not significantly different, whereas those with different superscript letters are significantly (P < 0.05) different as judged by Duncan’s multiple test df: degree of freedom, NaNO_3_- sodium nitrate, GW – NO_3_ contaminated ground water.

### 3.6 Histology

**Fig. 1:**
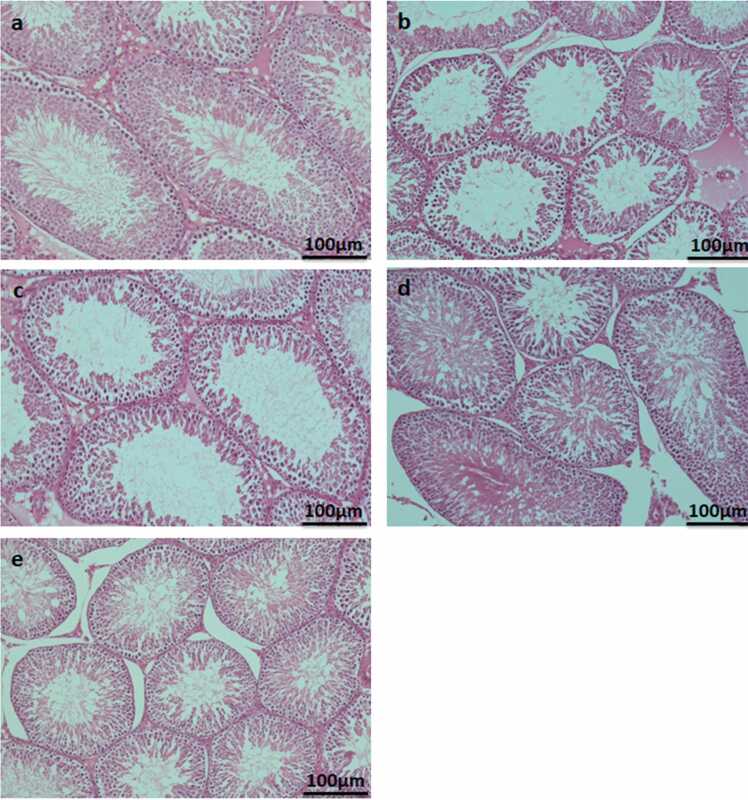
Photomicrographs of cross sections of testis showing seminiferous tubules of control with normal histological architecture (a). Note the loss of different stages of spermatocytes and reduced size of seminiferous tubules in rats treated with 100 mg/kg bw of NaNO_3_ (b) and NO_3_ contaminated ground water (c). Pretreatment of eugenol (100 mg/kg bw) prevented NaNO_3_ (d) and NO_3_ contaminated ground water (e) induced alterations in seminiferous tubules (H&E).

The seminiferous tubules of control testis showed normal histological architecture. However, degenerative spermatogenic epithelial cells were observed in seminiferous tubules of NaNO_3_ and NO_3_ contaminated ground water treated rats. The seminiferous tubules of 100 mg/kg bw of eugenol pretreated NaNO_3_ and NO_3_ contaminated ground water exposed testis showed normal histological details, similar to control (Fig. 1).

### 3.7 Counts of different categories of germ cells in stage VII of spermatogenesis in rats

There was a significant decrease in the number of type A spermatogonia, preleptotene spermatocytes, mid pachytene spermatocytes, round spermatids and elongated spermatids in NaNO_3_ and NO_3_ contaminated ground water treated rats compared to that of control rats. However, the count of preleptotene spermatocytes and mid pachytene spermatocytes of eugenol pretreated NO_3_ exposed rats was similar to that of control rats. The count of type A spermatogonia, round spermatids and elongated spermatids in eugenol pretreated NO_3_ exposed rats was significnatly higher than NaNO_3_ and NO_3_ contaminated ground water treated rats and lower than control rats (Table 8).

**Table 8:** Different categories of germ cells in stage VII of spermatogenesis of male rats.

Groups	Mean number of cells in tubular cross sections ± SE				
	Type A spermatogonia	Preleptotene spermatocytes	Midpachytene spermatocytes	Round spermatids	Elongated spermatids
**Control**	201.99±15.81^a^	123.40±16.27^a^	119.82±18.67^a^	573.98±56.86^a^	776.91±51.25^a^
**NaNO**_**3**_**(100 mg/kg bw)**	91.74±11.75^c^	43.41±7.05^b^	61.41±4.96^b^	57.24±3.63^c^	73.23±13.27^c^
**NO**_**3**_**contaminated ground water**	58.83±10.49^c^	32.32±6.36^b^	40.74±6.00^b^	14.58±1.41^c^	28.83±4.46^c^
**NaNO**_**3**_**+ Eugenol (100 mg/kg bw)**	170.03±8.45^ab^	109.98±11.17^a^	123.28±13.18^a^	377.43±24.17^b^	552.52±59.78^b^
**GW+ Eugenol (100 mg/kg bw)**	144.55±11.85^b^	101.81±10.65^a^	103.97±12.07^a^	308.19±45.87^b^	445.50±33.64^b^
**ANOVA F value** **df = (5, 24)**	23.70 P<0.0001	14.41 P<0.0001	9.31 P<0.0001	45.43 P<0.0001	67.89 P<0.0001

**Note**: Mean values with same superscript letters in the given column are not significantly different, whereas those with different superscript letters are significantly (P < 0.05) different as judged by Duncan’s multiple test df: degree of freedom, NaNO_3_- sodium nitrate, GW – NO_3_ contaminated ground water.

## 4 Discussion

Present study showed protective action of eugenol against NO_3_ induced oxidative stress in spermatozoa and testis. It was observed that NO_3_ exposure caused a significant decrease in relative weights of testis, epididymis, vas deferens and seminal vesicle, sperm motility, activities of testicular and spermatozoa CAT and SOD, concentration of serum testosterone, testicular 3β-HSD activity and number of type A spermatogonia, preleptotene spermatocytes, midpachytene spermatocytes, round spermatids and elongated spermatids. In addition, there was significant increase in percentage gain in body weight, number of abnormal spermatozoa and concentration of NO_2_ and MDA in testis and spermatozoa of NO_3_ exposed rats. Further, distorted histological architecture of seminiferous tubules, loss of cells of different stages of spermatogenesis and luminal spermatozoa were observed in seminiferous tubules of NO_3_ exposed rats compared to controls. These results clearly established the fact of NO_3_ induced reproductive toxicity in male rats.

It has been reported that NO_3_ is a potential endocrine disruptor and brings its effect through NO_2_ and NO [25]. Further, in biological system, inorganic NO_3_ and NO_2_ from endogenous or dietary sources are metabolized to NO. Nitric oxide is an important reactive nitrogen species (RNS) which affects the antioxidant system under its excess production and induces nitrosative stress [19]. The other highly reactive RNS includes, NO_2_, and peroxynitrite (ONOO^−^). The nitrosative stress is the result of increase in the content of RNS [26], [27]. It is evident that NO_2_ is capable of binding to hemoglobin and generates methemoglobin that causes a state of hypoxia by reducing the availability of methemoglobin to oxygen [23], [9]. It has been shown that NO_3_ exposure increases lipid peroxidation which was accompanied by inhibition of activities of SOD, CAT, GSH and glutathione peroxidase (GPx) in testis of rats and thereby attributed to testicular oxidative stress [1], [11], [8]. The present study strongly supports the above view wherein NO_3_ treatment resulted in increased concentration of NO_2_ in spermatozoa and testis. Concomitantly, there was decrease in the activities of SOD and CAT in spermatozoa and testis. Further, significant increase in the concentration of MDA; an indicator of lipid peroxidation in spermatozoa and testis of NO_3_ treated groups clearly proved the establishment of oxidative stress under NO_3_ treatment. These results strongly supported the fact that NO_3_ has potential to induce oxidative stress in spermatozoa and testis.

Further, earlier studies showed that spermatozoa are sensitive to oxidative stress which severely affects sperm motility [27]. The results of the present study revealed significant decrease in sperm motility and increase in sperm abnormality in NO_3_ treated groups compared to controls. These observations indicated that oxidative stress has a vital role in maintaining sperm quality. It has been shown that excess NO has direct influence in reducing the sperm motility by lowering the mitochondrial respiration and levels of ATP [55]. The result of the current work clearly established that NO_3_ induced oxidative stress caused decreased sperm motility and thereby affected the fertilization ability of spermatozoa.

The interesting part of the present study is the investigation on protective action of eugenol against NO_3_ induced oxidative stress in reproductive system of male rats. A pilot study was conducted where in three different concentrations *viz.,* 1, 10 and 100 mg/kg bw of eugenol were used [42]. Different parameters *viz.,* sperm motility, activity of testicular 3β-HSD and serum concentration of testosterone in 100 mg/kg bw of eugenol pretreated and NaNO_3_ exposed rats did not show significant alterations and were similar to controls. Therefore, the single dose of eugenol that is 100 mg/kg bw was used for the current investigation. The study demonstrated that pretreatment of 100 mg/kg bw of eugenol protected the male reproductive system against NO_3_ induced oxidative stress. Earlier studies showed aphrodisiac property of eugenol in Ayurvedic and Unani system of medicines in treatment of male sexual disorders [15], [51]. Further, eugenol was proved for its antioxidant properties [36]. In the present study, it was clear that majority of the parameters studied *viz.,* sperm motility, activities of SOD and CAT in testis and spermatozoa, concentrations of MDA and NO_2_ in testis and spermatozoa, activity of testicular 3β-HSD, serum concentration of testosterone and number of preleptotene spermatocytes and mid pachytene spermatocytes in 100 mg/kg bw of eugenol pretreated NO_3_ exposed groups were similar to controls. The near normal levels of NO_2_ and MDA and activities of antioxidant enzymes under eugenol pretreatment indicated the balanced antioxidant system despite NO_3_ exposure. The results of the present study are in line with the studies conducted by Barhoma [12] wherein pretreatment of eugenol prevented K_2_Cr_2_O_7_ induced alterations in renal antioxidant system of rats *viz.,* decreased activities of renal GSH and SOD and increased level of renal MDA. Similarly, pretreatment of eugenol prevented cisplatin induced increased MDA levels and decreased activities of GPx, GSH, SOD and CAT in rats [6]. These findings clearly suggested the antioxidant property of eugenol.

It has been shown that nitrosative/oxidative stress affect steroidogenesis in Leydig cells. High level of NO_2_ can interfere with steroidogenesis of Leydig cells and results in decreased production of testosterone [25], [27], [5] which in turn affects spermatogenesis as spermatogenesis is dependent on testosterone concentration. Further, excess production of NO_2_ can induce oxidative stress [40] and thereby decreases sperm motility and viability as spermatozoa are sensitive to the free radicals [45], [55]. However, eugenol pretreatment prevented the excess production of NO_2_ despite exposure to NO_3_ and thereby activities of antioxidant enzymes, lipid peroxidation, steroidogenesis, spermatogenesis and sperm motility were maintained to normal level. These findings clearly reveled the ameliorative action of eugenol on male reproductive system under NO_3_ exposure.

Further, it has been reported that eugenol inhibits lipid peroxidation both in *in vitro* and *in vivo* conditions [24], [36], [39]. It has been reported that phenolic antioxidants, including eugenol inhibit lipid peroxidation by acting as chain-breaking antioxidant [34] as well as enhancing the antioxidant enzyme activities [28], [43], [52]. Eugenol possess antioxidant property by virtue of its phenolic hydroxyl group in its structure that donates electrons to quench the free radicals. It also prevents the oxidation of Fe^2+^ by H_2_O_2_ in the Fenton reaction, which generates hydroxyl radicals involved in the initiation of lipid peroxidation [43]. A study by Said [47] showed that simultaneous treatment of eugenol in gentamicin-intoxicated rats decreased the level of lipid peroxidation and prevented the gentamicin-induced depletion of glutathione level as well as activities of SOD and CAT in kidney. Eugenol can decrease NO level due to its antioxidant property and radical scavenging activity and thereby prevent oxidative stress under NO_3_ exposure [24]. However, further studies are required to decipher the mode of action of eugenol in prevention of NO_3_ induced oxidative stress in male reproductive system. The current investigation was limited to the assessment of antioxidant property of eugenol in preventing NO_3_ induced male reproductive toxicity. Further, the study could not interpret the action of eugenol at cellular and molecular levels. In this line, future studies are required to assess the mode of action of eugenol in preventing NO_3_ induced reproductive toxicity.

## Ethics approval

The experimental procedures were in accordance with the guidelines of Institutional Animal Ethics Committee of University of Mysore, India (Reference number: UOM/IAEC/04/2018).

## Funding

This research received no specific grant from any funding agency in the public, commercial, private or not for profit sectors.

## CRediT authorship contribution statement

**Sulanaikanahalli Vadyappa Rajini:** Methodology, Investigation. **Halugudde Nagaraja Sarjan:** Visualization, Supervision. **Shivabasavaiah:** Resources, Project administration, Conceptualization.

## Declaration of Competing Interest

The authors declare that they have no known competing financial interests or personal relationships that could have appeared to influence the work reported in this paper.

## Data Availability

Data will be made available on request.
